# Binder-Free Spinel Co_2_CuO_4_ Nanosheet Electrodes with Cu-Driven Kinetic Enhancement for Alkaline OER Applications

**DOI:** 10.3390/ma19020301

**Published:** 2026-01-12

**Authors:** Abu Talha Aqueel Ahmed, Momin M. Mujtaba, Abu Saad Ansari, Sangeun Cho

**Affiliations:** 1 Division of System Semiconductor, Dongguk University, Seoul 04620, Republic of Korea; talhaphy@gmail.com; 2 Department of Physics, Maharaja Sayajirao Gaikwad Arts, Science and Commerce College, Malegaon-Camp, Malegaon 423203, India; mohammedmujtaba1318@gmail.com; 3 Nano Center Indonesia Research Institute, Puspiptek Street, South Tangerang 15314, Banten, Indonesia; saad@nano.or.id

**Keywords:** electrodeposition, Co_2_CuO_4_, nanosheets, Co_3_O_4_, OER, prolonged electrolysis

## Abstract

Developing electrocatalysts that are efficient and durable for the oxygen evolution reaction (OER) is essential for improving the energy efficiency of alkaline water splitting. Spinel-type transition-metal oxides have emerged as promising non-noble alternatives; however, their catalytic performance is often limited by sluggish charge transport and insufficient utilization of active sites. Herein, we present a systematic comparative study of electrodeposited Co_3_O_4_ (CO-300) and Cu-substituted Co_2_CuO_4_ (CCO-300) nanosheet films directly grown on Ni foam. Structural, morphological, and spectroscopic analyses reveal that Cu^2+^ integration into Co-oxide spinel lattice modifies the local electronic environment and produces a more open and interconnected nanosheet architecture, thereby enhancing conductivity and increasing the density of accessible redox-active sites. As a result, the optimized CCO-300 exhibits superior catalytic performance at higher current densities, along with a smaller Tafel slope (44 mV dec^–1^), a larger electrochemically active surface area (ECSA), and reduced charge-transfer resistance compared to CCO-300, indicating accelerated reaction kinetics and improved electron-ion transport. Furthermore, the multistep chronopotentiometry measurements and long-term stability tests over 100 h at current densities of 10 and 250 mA cm^–2^ highlight the excellent operational stability of the CCO-300 catalyst.

## 1. Introduction

The growing global energy demand, combined with the urgent need to mitigate carbon emissions, has intensified the search for sustainable and scalable energy technologies [1,2,3]. Although fossil-fuel-based systems continue to dominate the global energy supply, their finite availability and severe environmental consequences underscore the necessity of transitioning toward renewable alternatives [4,5]. To overcome these persistent limitations, hydrogen (H_2_) has emerged as a promising clean energy route owing to its high gravimetric energy density, carbon-free combustion products, and compatibility with decentralized power systems [6,7]. Among various hydrogen production pathways, alkaline water electrolysis is particularly attractive due to its operational simplicity, high purity output, and compatibility with intermittent renewable sources [8]. Nevertheless, the overall efficiency of water splitting remains severely constrained by the sluggish kinetics of the oxygen evolution reaction (OER), which involves complex multistep proton-coupled electron transfer processes [9]. Therefore, addressing this kinetic bottleneck is essential for reducing the required overpotential, improving energy efficiency, and enabling large-scale hydrogen production [10,11].

State-of-the-art OER catalysts, such as IrO_2_ and RuO_2_, exhibit excellent intrinsic activity; however, their scarcity, high cost, and poor long-term stability under strongly oxidizing alkaline conditions significantly hinder their widespread application [12,13,14]. Consequently, the development of earth-abundant, cost-effective, and durable OER electrocatalysts has become a central research focus. Transition-metal spinel oxides, particularly those based on Co, Ni, Fe, Cu and Mn, have gained significant attention due to their rich multivalent redox chemistry, structural robustness, and tunable electronic configurations [15,16,17,18,19,20]. Within this family, Co_3_O_4_ has emerged as a benchmark non-noble OER catalyst owing to its favorable Co^2+^/Co^3+^ redox pair and stable cubic spinel framework [21]. However, its relatively low electrical conductivity and limited exposure of catalytically active surface sites restrict its overall catalytic efficiency, motivating the development of effective strategies to enhance its intrinsic performance [22,23]. To address these issue, cationic substitution has proven to be an effective approach for modulating the electronic structure, optimizing the metal-oxygen covalency, and improving the redox flexibility of spinel oxides [24]. In particular, partial substitution of Co by Cu to form Co_2_CuO_4_ offers several notable advantages [25]. The incorporation of Cu introduces additional Cu^+^/Cu^2+^ redox couples, enhances p-d hybridization between metal cations and oxygen, and promotes improved charge-transfer kinetics [26]. Moreover, the mixed-metal environment increases the density of catalytically active octahedral sites, strengthens lattice-oxygen participation, and facilitates the adsorption and desorption of OER intermediates. The Cu substitution can also influence nucleation and morphological evolution during synthesis, promoting the formation of highly porous and interconnected nanosheet networks with shortened diffusion pathways and abundant accessible active sites, thereby enhancing overall OER performance. In addition, directly fabricating Co_2_CuO_4_ on conductive nickel foam ensures strong electrical contact, improved mechanical adhesion, and binder-free catalyst utilization, which are particularly beneficial for stable OER operation at high current densities. While these beneficial effects of Cu incorporation have been recognized in previous studies, their individual contributions are often intertwined with variations in synthesis conditions, morphology, or electrode configuration. As a result, the intrinsic role of Cu substitution in modulating electronic structure and redox behavior has not always been fully decoupled from these extrinsic factors. Therefore, controlled side-by-side evaluations using a well-defined Cu-free Co_3_O_4_ benchmark under identical synthesis and testing conditions are essential to more clearly elucidate the influence of Cu incorporation on active-site utilization, surface redox dynamics, and overall electrode performance during alkaline OER.

In this work, we present a systematic comparative study of electrodeposited Co_2_CuO_4_ (CO-300) and Cu-substituted Co_2_CuO_4_ (CCO-300) nanosheet electrode grown directly on Ni foam under identical testing conditions. By correlating the structural and morphological characteristics with comprehensive electrochemical measurements, we demonstrate that Cu incorporation imparts clear practical advantages during OER operation. The optimized CCO-300 electrode exhibits a significantly reduced overpotential and robust activity over a wide current range compared with pristine CO-300. This superior performance arises from the synergistic interaction between Cu and Co centers, which promotes efficient electron exchange, facilitates the generation of active oxygen species, and increases the density of electrochemically accessible sites. Furthermore, the binder-free nanosheet architecture ensures intimate contact with the Ni foam substrate, accelerates charge transport, and supports rapid oxygen-bubble release, thereby enabling sustained and stable OER operation. Overall, this study highlights CCO-300 as a highly efficient and durable non-noble spinel catalyst and offers a scalable, composition-tuned design strategy for advancing oxygen-evolution electrocatalysis.

## 2. Materials and Methods

### 2.1. Materials

**Figure 1:**
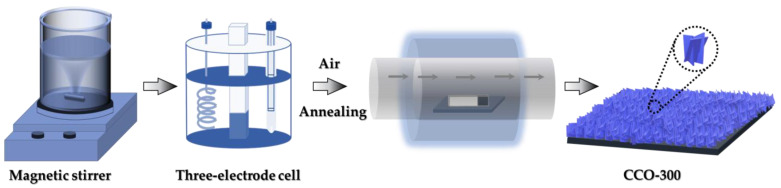
The schematic representation illustrates the overall growth and transformation pathway. It begins with the nucleation of metal hydroxide species on the conductive NF skeleton followed by anisotropic nanosheet growth driven by preferential facet orientation. Controlled thermal annealing then induces structural reorganization and crystallization, leading to the formation of interconnected porous nanosheet networks of Co_2_CuO_4_.

All reagents (Sigma-Aldrich, St. Louis, MO, USA) were of analytical grade and used as received without further purification. Copper(II) chloride dihydrate (CuCl_2_·2H_2_O, ≥98%) and cobalt(II) chloride hexahydrate (CoCl_2_·6H_2_O, ≥98%) were employed as metal precursors for the electrodeposition baths. Ammonium hydroxide solution (NH_4_OH, ≥99.99%) was used as a complexing/alkalizing agent. Potassium hydroxide (KOH, ≥85%) served as the electrolyte for electrochemical measurements. Acetone (CH_3_COCH_3_, ≥99.5%), ethanol (CH_3_CH_2_OH, ≥95%), and hydrochloric acid (HCl, 37%) were used for substrate surface pretreatment and cleaning. Commercial three-dimensional (3D) nickel foam (NF, thickness ~ 1.5 mm, porosity > 95%) was employed as the conductive substrate (Alantum, Seoul, Republic of Korea). Prior to the electrodeposition, NF pieces were cut into the desired geometric area (typically 1 × 5 cm^2^) and sequentially ultrasonicated in acetone, 3.0 M HCl, ethanol, and deionized water to remove surface oxides, organic impurities, and residual contaminants. The cleaned foams were then dried in an air oven and used immediately for catalyst deposition (Figure 1).

### 2.2. Synthesis of CO and CCO-300 Electrodes

The Co-based nanosheet electrode films were fabricated through a controlled electrodeposition-annealing procedure. For the preparation of Co_3_O_4_ (CO) electrodes, an aqueous electrolyte bath (50 mL) containing 8.0 mmol CoCl_2_·6H_2_O and NH_4_OH was prepared under continuous stirring. The cleaned 3D NF substrate served as the working electrode, while a Pt wire and a saturated calomel electrode (SCE) acted as the counter and reference electrodes, respectively. The electrodeposition was conducted using a constant potential of −1.0 V (vs. SCE) and the deposition duration was systematically varied to 150, 300, and 450 s to obtain the CO-150, CO-300, and CO-450 electrode films, respectively. After an electrodeposition, the electrode films were rinsed thoroughly with deionized water, dried overnight, and subsequently annealed in ambient air at 350 °C for 2 h to convert the hydroxide precursor into crystalline Co_3_O_4_ nanosheets. For the Cu-containing counterpart (i.e., Co_2_CuO_4_), a similar deposition method was adopted, except that the electrolyte contained both CoCl_2_·6H_2_O and CuCl_2_·2H_2_O (4.0 mmol) in appropriate stoichiometric ratio. Based on preliminary optimization, a deposition time of 300 s yielded the best nanosheet morphology and electrochemical response, this sample is referred to as CCO-300. The deposited Cu-Co hydroxide electrode film was washed, dried, and annealed under identical conditions to form spinel Co_2_CuO_4_. The mass loading of the electrodeposited films was carefully measured and found to be approximately 0.76 mg (CO-300) and 0.83 mg (CCO-300).

### 2.3. Material Characterization

The morphological, structural, and surface chemical properties of the synthesized electrodes were comprehensively examined using various analytical techniques. Raman spectroscopy was performed using a 514 nm laser excitation source to probe lattice vibrational modes and confirm the structural integrity of the spinel framework using LabRam Aramis spectrometer (Horiba Jobin Yvon, Tokyo, Japan). The morphological features and nanosheet architecture were investigated using field-emission scanning electron microscopy (FESEM, JSM-6701F, JEOL, Tokyo, Japan). Elemental composition and spatial distribution of O, Cu, and Co across the nanosheet films were further examined through energy-dispersive X-ray spectroscopy (EDS) and corresponding elemental mapping. The crystallographic phases and lattice features were analyzed by X-ray diffraction (XRD, Rigaku Smartlab instrument, Akishima, Japan) using a Cu Kα radiation source (λ = 1.5406 Å). Diffraction patterns were recorded in the 2θ range of 20–80° to identify spinel phase formation. Surface chemical states and oxidation environments were analyzed by X-ray photoelectron spectroscopy (XPS, ULVAC PHI 5000 VersaProbe, Chigasaki, Japan). High-resolution spectra of Co 2p, Cu 2p, O 1s, and C 1s regions were collected, calibrated against adventitious carbon (C 1s at ~285 eV), and deconvoluted using a Gaussian fitting protocol.

### 2.4. Electrochemical Test

#### Catalytic OER Activity

The electrochemical OER performances of the synthesized electrode films were evaluated using a three-electrode configuration on a VersaSTAT workstation (Berwyn, PA, USA). The as-prepared CO-150, CO-300, CO-450 and CCO-300 nanosheet films on Ni foam served as the working electrodes, with a Pt wire and SCE functioning as the counter and reference electrodes, respectively. All measurements were performed in 1.0 M KOH electrolyte at room temperature under ambient conditions. Linear sweep voltammetry (LSV) was carried out in the potential range appropriate for alkaline OER at a scan rate of 1.0 mV s^−1^. The obtained potentials were converted to the reversible hydrogen electrode (RHE) scale using:

*E*_RHE_ = *E*_SCE_^∘^ + (0.059 × *pH*) + *E*_SCE_, (1)

where *E*_SCE_^∘^, *E*_RHE_, and *E*_SCE_ are the standard potential of SCE at room temperature, potential in RHE scale, and potential in SCE scale, respectively. Internal ohmic losses were minimized by *JRs* (i.e., *iRs*) compensation, where the series resistance (*Rs*) was extracted from the high-frequency intercept of the Nyquist plot and The Tafel slope was derived from the LSV data by fitting the linear region of the “*η* versus log(*J*)” relationship, which can be expressed as follows:

*E*_RHE_ (*JRs-correction*) = *E*_RHE_ − (*J* × *R*s), (2)

*η* = *E*_RHE_ (*JRs-correction*) − 1.23, (3)

*η* = *a* + *b* × log(*J*), (4)

where *a* is the Tafel slope and *b* is an arbitrary constant. The chronopotentiometry was employed to scrutinize the durability over the prolonged electrolysis in an alkaline condition. The potential response at each current level was monitored over extended durations to evaluate mechanical adhesion, structural integrity, and catalytic durability. All measurements were repeated using independently prepared electrodes to ensure reproducibility (Figure S1). The electrochemical impedance spectroscopy (EIS) was performed at a fixed overpotential using an applied AC signal amplitude of 10 mV over the frequency range from 0.01 to 100 kHz. The resulting Nyquist spectra were fitted using an equivalent circuit model to obtain the charge-transfer resistance (*Rct*). The electrochemically active surface area (*ECSA*) was estimated through non-faradaic cyclic voltammetry (CV) measured at varying scan rates (10–100 mV s^−1^) within a potential window free of redox features. The slope of the capacitive current versus scan rate plot provided the double-layer capacitance (*C_DL_*), which was used as a proportional measure of *ECSA*.

## 3. Results

### 3.1. Crystallographic and Bonding Properties of the Electrode Films

**Figure 2:**
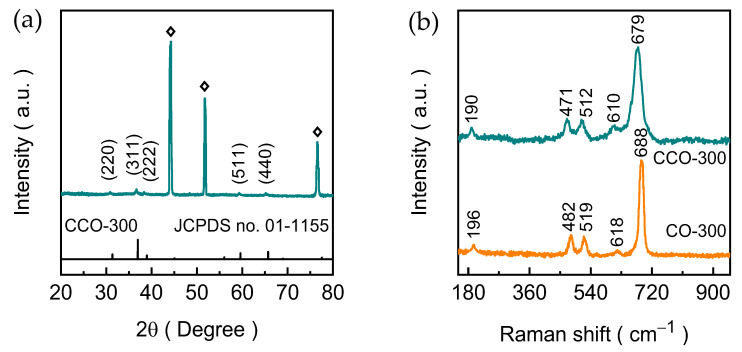
(**a**) XRD spectra of CCO-300 electrode film along with the JCPDS card and (**b**) Raman spectra of CO-300 and CCO-300 electrode films.

The crystallographic features and phase evolution of the electrodeposited CO-300 and CCO-300 electrode films were thoroughly examined using the XRD technique, and the corresponding diffraction patterns are presented in Figure 2a. All samples exhibit three intense reflections located at approximately 44.32°, 51.71°, and 76.52°, which originate from the (111), (200), and (220) planes of the underlying Ni foam substrate, a characteristic and unavoidable contribution due to its high metallic reflectivity. The diffraction pattern of the CO-300 electrode (Figure S2) shows well-defined XRD peaks at 2θ ≈ 31.23°, 36.81°, 59.32°, and 65.25°, indexed to the (220), (311), (511), and (440) planes of the cubic spinel Co_3_O_4_ phase (JCPDS No. 76-1802) [27]. These peaks confirm the successful transformation of the electrodeposited hydroxide precursor into a highly crystalline spinel oxide during thermal annealing. Upon incorporation of Cu, the diffraction pattern of the CCO-300 electrode displays a diffraction peak located at 2θ ≈ 30.87°, 36.65°, 38.34°, 59.33°, and 65.17°, consistent with the characteristic (220), (311), (222), (511), and (440) planes of cubic Co_2_CuO_4_ (JCPDS No. 01-1155) [28]. The close similarity in peak positions between CO-300 and CCO-300 electrodes is expected due to the structural compatibility of the two spinel lattices. However, a slight broadening and marginal shift in peak position are observed for CCO-300, which is a localized lattice distortion resulting from the substitution of Cu^2+^ into Co-based octahedral sites, which slightly modifies the unit-cell environment [29]. This macrostrain effects are frequently associated with enhanced redox flexibility and improved catalytic responsiveness in mixed-metal spinel materials. Notably, no secondary phases or other mono/binary oxides were detected, confirming the phase purity and successful integration of Cu into the spinel framework.

Raman spectroscopy was employed to further validate the lattice structure and vibrational characteristics of the electrodeposited CO-300 and CCO-300 electrode films. Figure 2b shows the corresponding Raman spectra of the CO-300 and CCO-300 electrode films. The CO-300 electrode exhibits well-defined vibrational modes located at approximately 196, 482, 519, 618, and 688 cm^−1^, which correspond to the F_2g_^3^, E_g_, F_2g_^2^, F_2g_^1^, and A_1g_ phonon modes of the cubic spinel Co_3_O_4_ framework [30]. These vibrations arise from symmetric and asymmetric stretching vibrations within the Co-O polyhedral units and confirm the formation of a long-range ordered Co_3_O_4_ lattice [31,32]. Although the CCO-300 electrode displays a similar set of vibrational bands, the principal vibrational modes are slightly broadened and red-shifted compared to the CO-300 electrode. The modest red-shift indicates a variation in metal-oxygen bond strength, consistent with partial substitution of Co by Cu in the octahedral sites of the spinel lattice [33]. This cation exchange modifies the local coordination environment and increases lattice polarizability, a characteristic feature commonly observed in mixed-metal spinel oxides. Moreover, the broadening of the high-frequency A_1g_ mode suggests enhanced structural disorder and increased oxygen-vacancy concentration, both of which are known to facilitate redox activity and improve charge-transfer properties during the OER process [34,35]. The absence of any additional peaks corresponding to impurity phases such as CoO or CuO further supporting the XRD conclusion that the electrodeposition-annealing process yields phase-pure spinel oxides. The vibrational fingerprints of both samples confirm that Cu incorporation preserves the overall spinel symmetry while subtly modifying the local bonding network, which directly correlate with the change in electrocatalytic performance.

### 3.2. Morphological and Compositional Properties of the Electrode Films

**Figure 3:**
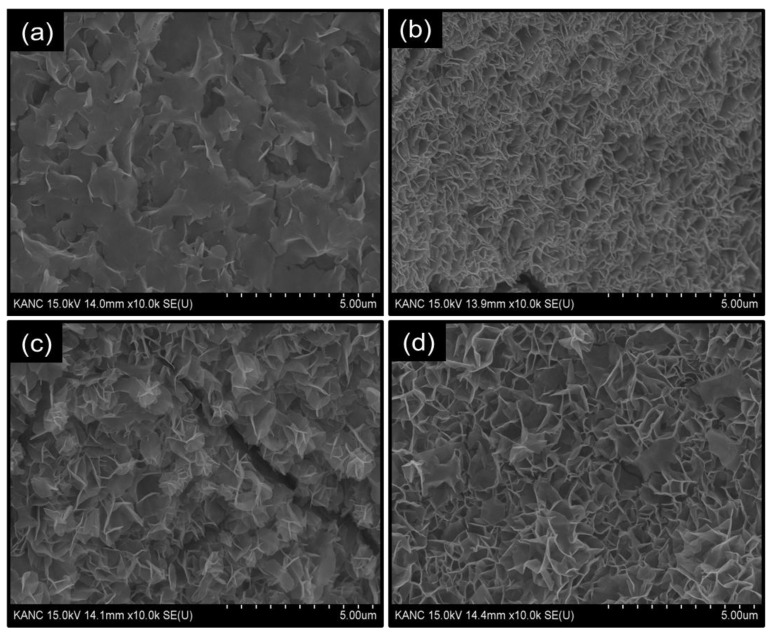
FESEM images Co_3_O_4_: (**a**) CO-150, (**b**) CO-300, (**c**) CO-450, and (**d**) CCO-300 electrode films recorded to help understand the structural evolution with electrodeposition time.

The structural evolution of the Co_3_O_4_ electrode films with electrodeposition time was examined using FESEM. Figure 3a shows the FESEM image of CO-150, which displays an initial population of thin and loosely arranged nanosheets fragments that partially cover the Ni foam skeleton, reflecting the early stage of nucleation-driven growth. These early-stage sheets appear relatively smooth and sparsely distributed, reflecting limited nucleation and insufficient lateral growth at short deposition times. Upon extending the deposition, the FESEM image of CO-300 (Figure 3b) showcase the nanosheets become significantly more abundant, forming a continuous and highly interwoven network. The 2D nanosheets develop pronounced wrinkling and curvature, producing a more open and texturally rich morphology. This configuration might provide numerous exposed edges and interconnected void channels, which are advantageous for electrolyte diffusion and electrochemical utilization of active sites. However, for the CO-450 electrode the FESEM image (Figure 3c) the nanosheet morphology undergoes a significant transformation. Instead of further lateral expansion, the nanosheets begin to stack vertically, generating compact, flower-like aggregates composed of overlapped and clustered nanosheets. This overgrowth leads to a congested and partially collapsed surface with reduced inter-sheet spacing, while localized micro-cracks appear due to increased internal stress within the densely packed film. The excessive stacking and morphological congestion at 450 s diminish the effective porosity and hinder electrolyte accessibility, elucidating the inferior electrochemical response compared to CO-300 (Figure S3). Following this optimized condition, CCO-300 was electrodeposited at the identified ideal duration of 300 s to obtain its corresponding nanosheet architecture. As shown in Figure 3d, the CCO-300 electrode preserves the overall 2D nanosheet framework of CO-300; however, the introduction of Cu results in a noticeably more open and interconnected morphology. The nanosheets exhibit slightly larger lateral dimensions and sharper edges with well-defined inter-sheet spacing compared to the CO-300 electrode, along with broader inter-sheet channels. These structural changes arise from Cu-induced modifications in nucleation and surface-energy distribution, leading to a more dispersed and less compact nanosheet assembly. The improved openness and uniform thinness of the sheets provide greater accessibility to active surfaces and facilitate rapid electrolyte infiltration and gas removal during OER, ultimately offering a better electrochemically advantageous architecture than pristine CO-300.

### 3.3. Chemical Composition and Bonding States of Electrodes Films

**Figure 4:**
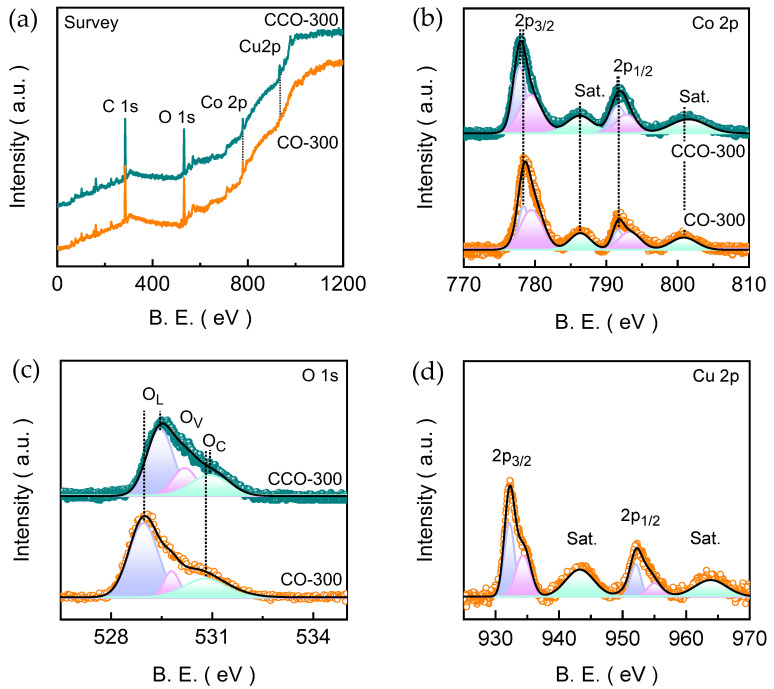
(**a**) XPS Survey spectra and high-resolution (**b**) Co 2p, (**c**) O 1s, and (**d**) Cu 2p emission spectra of CO-300 and CCO-300 electrode films. All the high-resolution XPS emission spectra were fitted using Gaussian curve fitting model.

The elemental composition of the electrodeposited films was examined using the EDS technique. For the CO-300 electrode film (Figure S4a), the EDS spectrum displays strong and well-defined signals corresponding to Co and O, confirming the successful formation of the cobalt oxide phase. The accompanying elemental atomic percentage shows that both Co and O are presented in stoichiometric ratio, which is consistent with the spinel Co_3_O_4_ structure. Whereas the EDS spectrum of CCO-300 reveals distinct peaks for Cu, Co, and O, consistent with the stoichiometry of the targeted Co_2_CuO_4_ spinel phase (Figure S4b). The quantitative analysis yields a Co:Cu atomic ratio close to 2:1, verifying the intended composition. To gain deeper insight into the surface chemistry and oxidation states of the transition-metal constituents in the electrodeposited films, the XPS was carried out for both CO-300 and CCO-300 electrodes (Figure 4). The survey spectra confirm the presence of Co and O in CO-300 (Figure 4a), whereas CCO-300 additionally exhibits distinct Cu signals, verifying the successful incorporation of Cu into the spinel lattice without the detection of any additional species. The high-resolution Co 2p spectrum of CO-300 (Figure 4b) shows two primary spin-orbit peaks related to the Co 2p_3/2_ (778.37 eV) and Co 2p_1/2_ (791.90 eV) emissions along with the well-resolved shake-up satellites marked as Sat. (786.35 and 800.93 eV). The deconvolution of these Co 2p_3/2_ (778.42 and 779.48 eV) and Co 2p_1/2_ (791.59 and 793.33 eV) degenerate state leads to the total of six peaks in the emission spectrum. These overlapping deconvoluted signals of the spin-orbit component aroused from the Co^2+^ and Co^3+^ species, which is consistent with typical cobalt spinel oxides [36].

The high-resolution O 1s spectrum of CO-300 (Figure 4c) shows three characteristic deconvoluted emission signals marked as O_L_ (528.96 eV), O_V_ (529.78 eV), and O_C_ (530.80 eV). These emission peaks originated from the lattice oxygen, oxygen vacancies, and chemisorbed oxygen or water linked with surface hydroxyl species [37]. Notably, the Co 2p spectrum of CCO-300 (Figure 4b) maintains the same spinel characteristics as that of the pristine CO-300; however, the deconvoluted peaks reveal a slight negative shift in both Co 2p_3/2_ and Co 2p_1/2_ emissions. This could be a result of enhanced electron density around Co centers owing to Cu incorporation, indicating strengthened metal-oxygen covalency and modified electronic structure favorable for OER [38]. The Cu 2p spectrum of the CCO-300 (Figure 4d) also shows two characteristic spin-orbit peaks related to the Cu 2p_3/2_ (932.21 eV) and Cu 2p_1/2_ (952.36 eV) emissions accompanied by two shake-up satellites (943.29 and 963.85 eV), which were further deconvoluted and resolving into a total of six peaks. The former peaks located at 932.21 and 952.16 eV correspond to the Cu^+^ state, whereas the emission peaks located at 934.43 and 954.81 eV correspond to the Cu^2+^ state of Cu [39]. This dual-valency enhances charge redistribution within the spinel lattice and supplies additional redox-active centers. The intensity of O 1s emission is almost similar to that of the CO-300 electrode film; however, the spectrum shows a slightly intense defect-related oxygen component. These vacancy-related states are known to facilitate the charge-transfer kinetics and promote the formation of higher-valent Co species. To further quantify the electronic structure differences, the relative ratios of Co^2+^/Co^3+^ and defect-related oxygen to lattice oxygen (O_V_/O_L_) were estimated from the fitted high-resolution XPS spectra. Compared to the CO-300 electrode, the CCO-300 electrode exhibits a higher fraction of mixed-valence metal centers and an increased O_V_/O_L_ ratio (from 0.32 to 0.35), reflecting the enhanced electronic flexibility and a greater density of oxygen defects induced by Cu incorporation. The alteration in electronic and defect-structure helps to facilitate the charge redistribution and OER intermediate adsorption, contributing to the improved electrochemical performance of the CCO-300 electrode. Nonetheless, the presence of mixed valence states of Co^2+^/Co^3+^ and Cu^+^/Cu^2+^ species confirm the formation of Co_3_O_4_ and Co_2_CuO_4_ spinel phases.

### 3.4. Electrochemical OER Properties of the Electrode Films

**Figure 5:**
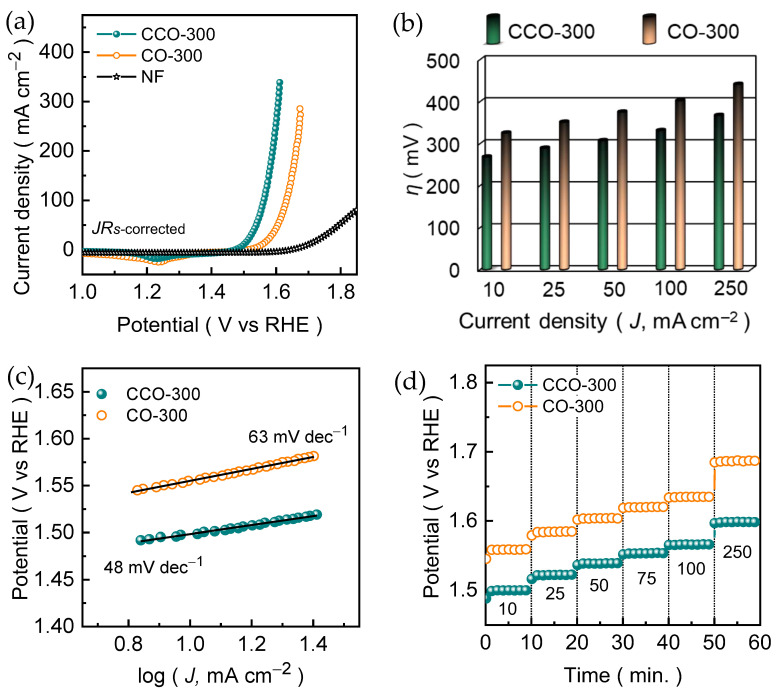
Electrocatalytic OER activity of CO-300 and CCO-300 catalysts examined in an alkaline 1.0 M KOH condition. Comparative (**a**) LSV curves, (**b**) Overpotential, (**c**) Tafel slopes, and (**d**) Potential as a function of current density.

The OER activity of the CO-300 and CCO-300 nanosheet electrode films was then evaluated in 1.0 M KOH using a standard three-electrode configuration. Figure 5a shows the *JR*-corrected LSV curves. The CCO-300 nanosheet electrode achieves a current density of 10 mA cm^−2^ at an overpotential of 268 mV, whereas the CO-300 nanosheet electrode requires a substantially higher potential of 324 mV to reach the same current. The clear shift of the polarization curve toward lower overpotential values demonstrates that Cu incorporation into the Co_3_O_4_ spinel markedly enhances the OER activity. This value is even comparative to the various recently reported Cu/Co-based metal oxide catalyst in alkaline KOH medium (Table S1). At a higher current density, the performance gap becomes more pronounced across the entire measured current density range (Figure 5b), CCO-300 electrode consistently exhibits steeper current rise and lower operating potentials compared to the CO-300 electrode, indicating superior charge-transfer efficiency and more effective utilization of surface-active sites under the alkaline OER conditions. The CCO-300 and CO-300 electrodes achieves the current densities of 25, 50, 75, 100, and 250 mA cm^−2^ at an overpotential of 289, 307, 320, 331, and 367 mV and 351, 375, 391, 402, and 441 mV, respectively. Moreover, the NF LSV was also recorded for comparison under the identical experimental conditions. The NF exhibits negligible OER activity, requiring significantly higher overpotential to reach even modest current densities. This poor activity arises from its limited intrinsic catalytic capability and the scarcity of accessible redox-active sites, confirming the observed OER enhancement in CO-300 and CCO-300 originates from the deposited spinel nanosheet films rather than the underlying substrate.

To further elucidate the intrinsic catalytic kinetics of the CO-300 and CCO-300 electrode films, Tafel analyses were performed based on the corresponding polarization curves (Figure 5c). The Tafel slopes were extracted from the linear region of the low-to-moderate overpotential range, where the current response is dominated by kinetic control and mass-transport effects are minimal. The CCO-300 nanosheet electrode exhibits a significantly lower Tafel slope of 44 mV dec^−1^ compared to the CO-300 electrode (63 mV dec^−1^), reflecting the faster reaction kinetics and a more efficient catalytic pathway for oxygen evolution. In alkaline media, OER typically proceeds through a multi-step proton-coupled electron-transfer sequence involving the adsorption of OH^−^ ions, the formation of surface M-O and M-OOH intermediates, and their subsequent conversion to molecular O_2_. The reduced Tafel slope of CCO-300 nanosheet electrode compared to CO-300 electrode suggest that Cu incorporation effectively improves the electrochemical reaction kinetics during these steps, modulate the electron density around cobalt centers facilitating the transition from Co^2+^ to catalytically active Co^3+^ during the reaction cycle [39]. Moreover, the open and interconnected nanosheet architecture of CCO-300 nanosheet electrode provides more accessible redox-active sites (Figure S5), reduces the charge-transfer resistance (Figure S6a, Table S2), and minimizes mass-transport limitations (Figure S6b), which enables the rapid intermediate formation and turnover.

The reduced Tafel slope of CCO-300 nanosheet electrode, together with its enhanced electrochemical performance, suggests that a kinetically more favorable OER pathway is induced by Cu incorporation. The accelerated kinetics can be attributed to Cu-driven electronic modulation within the spinel lattice, which facilitates interfacial electron transfer and stabilizes key OER intermediates. In addition, the presence of defect-related oxygen species identified by XPS indicates the modified metal-oxygen bonding environments that are known to promote adsorption and turnover of oxygenated intermediates. Moreover, to evaluate intrinsic catalytic activity independent of surface-area effects, the OER activity was further evaluated on an *ECSA*-normalized basis (Figure S6c). The *ECSA*-normalized current density of CCO-300 nanosheet electrode remains higher (~6-fold) than that of CO-300 electrode at the same potential of 351 mV, indicating that Cu incorporation improves intrinsic activity in addition to increasing electrochemically accessible surface area. This analysis is consistent with the reduced Tafel slope and lower charge-transfer resistance of CCO-300 nanosheet electrode, which signify accelerated intrinsic OER kinetics. Thereafter, the stepwise chronopotentiometric curves were recorded to further understand the insight into the dynamic OER behavior of the CO-300 and CCO-300 nanosheet electrodes across a wide current range. Figure 5d shows the obtained voltage-step curves of CO-300 and CCO-300 nanosheet electrodes at various current densities (10 to 250 mA cm^−2^). Both electrodes exhibit stable potential plateaus at each applied current density rate, indicating the good operational integrity under varying OER conditions. Interestingly, the consistently lower potentials of CCO-300 nanosheet electrode across the entire current sequence compared to the CO-300 electrode clearly demonstrate its superior catalytic efficiency, improved operational tolerance, and enhanced adaptability to varying current demands. The stable and nearly instantaneous potential stabilization at each step highlights the robust interfacial contact between the catalyst electrode and Ni foam, as well as the favorable electrolyte accessibility enabled by its open network and interconnected nanosheet architecture.

**Figure 6:**
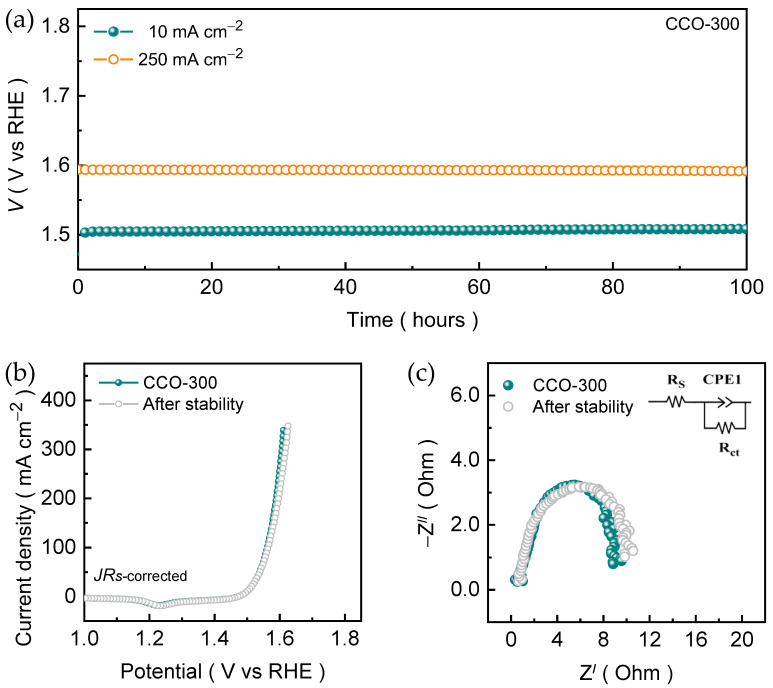
(**a**) Electrocatalytic OER stability curves of CCO-300 electrode film examined in an alkaline 1.0 M KOH condition at a current density of 10 and 250 mA cm^−2^. A comparative post-stability measured (**b**) LSV and (**c**) EIS curves.

The operational durability of the optimized CCO-300 nanosheet electrode film was assessed using the extended long-term chronopotentiometric measurements under alkaline KOH medium. Figure 6a shows the prolonged chronopotentiometric stability curves measured at a current density of 10 and 250 mA cm^−2^. At the low operating current density of 10 mA cm^−2^, the CCO-300 nanosheet electrode exhibits a brief initial potential change during the initial testing followed by the stable potential plateau that persists throughout the entire stability duration of 100 h. This initial equilibration is typical for OER catalysts and is associated with surface wetting, activation of redox-active sites, and stabilization of the electrode-electrolyte interface, which results in the partial surface conversion into oxyhydroxide phase upon electrooxidation process in an alkaline KOH medium. After this short transient stage, no further potential change is observed, confirming the structural and electrochemical robustness of the nanosheet architecture. The post-stability measured Raman spectrum (Figure S7a) of the CCO-300 electrode reveals the appearance of characteristic vibrational features attributable to CoOOH species, indicating the partial surface transformation of the spinel under anodic polarization and further supported by post-stability measured EDS spectrum (Figure S7b). The spectrum reveals the continued presence of Co and Cu, accompanied by a slight increase in the relative O signal intensity, which is consistent with surface oxidation or hydroxylation during OER and further supports the formation of CoOOH species. Even under the higher current density of 250 mA cm^−2^, where vigorous oxygen bubble evolution typically accelerates catalyst degradation, the CCO-300 nanosheet electrode maintains a remarkably steady potential with negligible deviation over 100 h. This exceptional stability indicates that the open, interconnected nanosheet network effectively mitigates bubble accumulation and minimizes concentration polarization, thereby ensuring uninterrupted electron and ion transport during extended anodic polarization. The post-stability LSV and EIS measurements were carried to verify retention of the catalytic activity. The post-stability measured polarization curve (Figure 6b) nearly overlaps with the initial one, exhibiting an insignificant change in the overpotential after the prolonged testing. Further, the charge-transfer resistance extracted from Nyquist plots (Figure 6c) remains almost unchanged after 100 h of continuous operation. The preservation of both kinetic and interfacial parameters strongly confirms that the intrinsic catalytic properties of the CCO-300 nanosheets remain intact, with no evidence of deactivation or loss of electrochemical accessibility.

## 4. Conclusions

In summary, this work provides a comprehensive evaluation of electrodeposited Cu-Co spinel nanosheet electrode films, demonstrating that cation modulation and nanoscale structural engineering synergistically enhance oxygen evolution reaction (OER) performance in alkaline media. The incorporation of Cu plays a decisive role in tailoring both the electronic structure and the morphological characteristics of the spinel lattice. The introduction of Cu^2+^ ions modifies the local coordination environment within the Co-O framework, leading to enhanced electronic delocalization and facilitated charge transfer across the electrode–electrolyte interface. These electronic effects, combined with the formation of a more open and interconnected nanosheet architecture, endow the CCO-300 electrode with increased exposure of redox-active sites, reduced transport resistance, and more efficient turnover of OER intermediates compared to pristine CO-300 electrode. As a result, the CCO-300 nanosheet electrode delivers a low overpotential of 268 mV at 10 mA cm^−2^ and maintains superior performance across higher current densities, consistently outperforming CO-300 (324 mV at 10 mA cm^−2^). The markedly smaller Tafel slope of 48 mV dec^−1^ and higher non-Faradaic capacitance further affirm the accelerated interfacial kinetics and increased electrochemically accessible surface area, enhanced conductivity, and favorable reaction pathways within the modified spinel lattice. Moreover, the optimized CCO-300 exhibits outstanding durability under operationally relevant conditions. The catalyst maintains stable potentials during multistep current operation and demonstrates exceptional long-term stability over 100 h at both 10 and 300 mA cm^−2^, with negligible degradation. Post-stability LSV and EIS analyses confirm the retention of intrinsic activity and preservation of the nanosheet architecture, which further validates the robustness of the directly grown electrode design. These findings demonstrate the CCO-300 nanosheet electrode as an efficient and durable OER catalyst and highlight the broader promise of cation-engineered spinel oxides for high-performance OER applications.

## Data Availability

The original contributions presented in this study are included in the article/Supplementary Material. Further inquiries can be directed to the corresponding author.

## Supplementary Materials

The following supporting information can be downloaded at https://www.mdpi.com/article/10.3390/ma19020301/s1. Figure S1: Reliability data of (a) Co_3_O_4_ and (b) Co_3_S_4_ catalysts measured for the series of sample in the same experimental conditions; Figure S2: XRD spectra of the CO-300 electrode film along with the standard JCPDS reference card number 76-1802; Figure S3: LSV curves recorded at a scan rate of 1.0 mV s^−1^ and (b) Tafel plots of the CO-150, CO-300, and CO-450 electrode films measured in alkaline KOH condition; Figure S4: EDS spectra of the (a) CO-300 and (b) CCO-300 electrode films; Figure S5: Non-Faradaic CV curves of the (a) CO-300 and (b) CCO-300 electrode films measured at different scan rates. (c) Non-Faradaic current densities as a function of scan rate obtained at 0.05 V (vs. SCE) to estimates the *C_DL_* and *ECSA*; Figure S6: (a) Nyquist impedance curves along with the tank circuit, (b) Mass activity plots, and (c) *ECSA*-normalized LSV curves for the CO-300 and CCO-300 electrode films; Figure S7: Post-stability measured (a) Raman and (b) EDS spectrum for the CCO-300 electrode film; Table S1: The electrochemical OER performance of our optimized CCO-300 electrode film and other metal oxide-based catalyst in alkaline electrolyte medium at 100 mA cm^−2^ (Refs [40,41,42,43,44,45,46,47,48,49,50,51,52] are included in the Supplementary Materials); Table S2: Fitted electrochemical EIS parameters for the CO-300 and CCO-300 electrodes obtained from Nyquist plots using the equivalent circuit model shown in Figure S6a. The table summarizes the solution resistance (*R*s), charge-transfer resistance (*R*ct), and constant phase element (CPE) parameters extracted from nonlinear least-squares fitting. All EIS measurements were performed in 1.0 M KOH under identical conditions.

## Abbreviations

The following abbreviations are used in this manuscript:

H_2_	Hydrogen
CuCl_2_·2H_2_O	Copper(II) chloride dihydrate
CoCl_2_·6H_2_O	Cobalt(II) chloride hexahydrate
CO	Co_3_O_4_
OER	Oxygen evolution reaction
XRD	X-ray diffraction
*C_DL_*	Double-layer capacitance
EDS	Energy-dispersive X-ray spectroscopy
FESEM	Field-emission scanning electron microscopy
XPS	X-ray photoelectron spectroscopy
CCO-300	Co_2_CuO_4_
3D	Three-dimensional
NF	Nickel foam
*R*ct	Charge transfer resistance
EIS	Electrochemical impedance spectroscopy
SCE	Saturated calomel electrode
*η*	Overpotential
CV	Cyclic voltammetry
RHE	Reversible hydrogen electrode
LSV	Linear sweep voltammetry
*ECSA*	electrochemically active surface area
