# Supplementary material for: Binder-Free Spinel Co2CuO4 Nanosheet Electrodes with Cu-Driven Kinetic Enhancement for Alkaline OER Applications
Source: Materials (Basel). 2026 Jan 12;19(2):301. doi: 10.3390/ma19020301 (PMC12842824; doi:10.3390/ma19020301)
Supplement: Supplementary file 1 [file materials-19-00301-s001.zip › materials-4046170-supplementary.pdf]

Supporting Information

Binder-Free Spinel Co_2CuO_4 Nanosheet Electrodes with Cu-Driven Kinetic Enhancement for Alkaline OER Applications

Abu Talha Aqueel Ahmed [1], Momin M. Mujtaba [2], Abu Saad Ansari [3] and Sangeun Cho [1,*]

[1] Division of System Semiconductor Science, Dongguk University, Seoul 04620, Republic of Korea

[2] Department of Physics, Maharaja Sayajirao Gaikwad Arts, Science and Commerce College, Malegaon-Camp, Malegaon 423203, India

[3] Nano Center Indonesia Research Institute, Puspiptek Street, South Tangerang, Banten 15314, Indonesia

Corresponding Author: sangeun.c@dongguk.edu

Table S1. The electrochemical OER performance of our optimized CCO-300 electrode film and other Cu/Co–based metal oxide catalyst in alkaline KOH electrolyte medium at 100 mA cm^{-2}.

No	Catalyst film	Overpotential (mV) @100 (mA cm^{-2})	Tafel slope (mV dec $^{-1}$)	Stability at *J* (*J* in mA cm^{-2})	Supporting Reference
1	Ni-Fe-OH@Ni_3S_2/NF	165	93	50 h@100	[40]
2	$P_{3.85}$-CCO/NF	250	27	30 h@10	[41]
3	$Ni_{50}Fe_{50}$-DAT	300	–	72 h@100	[42]
4	S-r-CCO	248	37.7	100 h@10	[43]
5	NiFeSedO-NiNF	247	54	~8 h@100	[44]
6	Fe^0-Ni_xS_y/NF	186	92	10 h@1.47 V	[45]
7	$CuCo_2O_4$	346	75.9	-	[46]
8	$CuCe_{0.5}Co_{1.5}O_x$	294	57.5	100 h	
9	CFP NPs	294	50	70 h@100	[47]
10	$Ni_3Fe(OH)_9$/NF	370	28	10 h@100	[48]
11	$CuCo_2O_4$@CNFs	273	82.8	12 h@10	[49]
12	CoS/MoS_2 heterostructure	294	31	100 h@100	[50]
13	NiFe-NF	290	50.1	30 h@100	[51]
14	N−CuCo2O4@N−C	260	90.1	28 h@1.59 V	[52]
**15**	**CCO-300 nanosheets**	**331**	**48**	**10@100**<br>**250@100**	**Present Work**

Supporting Figures

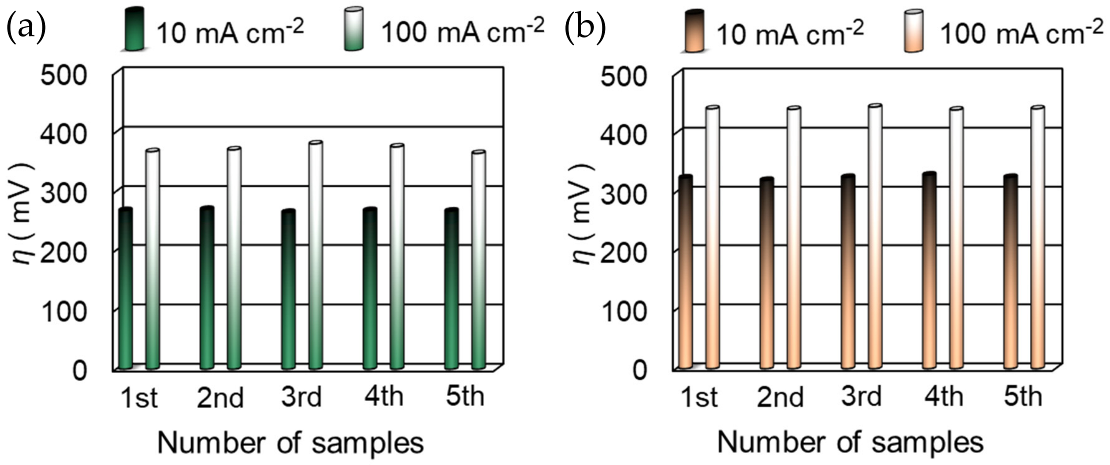

Figure S1. Reliability data of (**a**) CCO-300 and (**b**) CO-300 catalysts measured for the series of sample in the same experimental conditions.

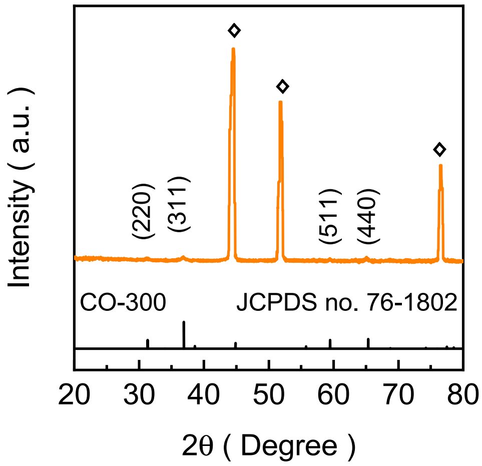

Figure S2. XRD spectra of the CO-300 electrode film along with the standard JCPDS reference card number 76-1802.

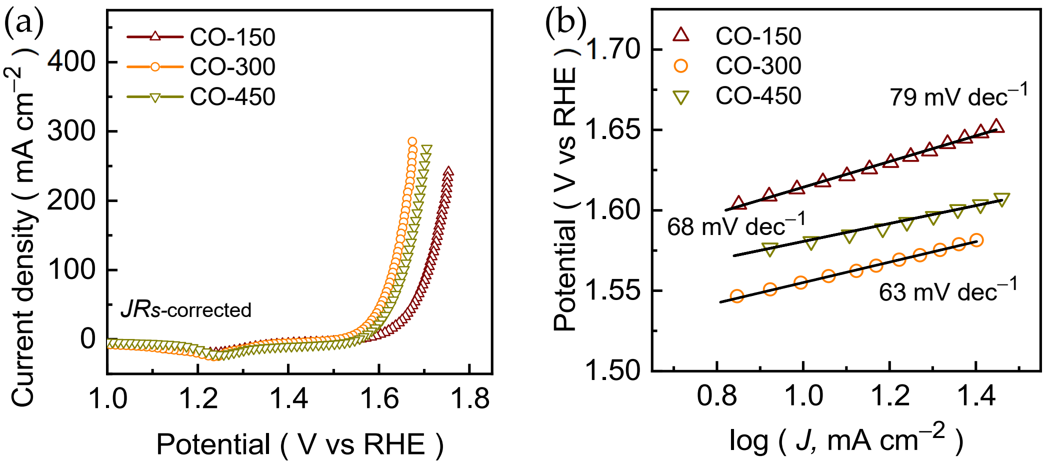

Figure S3. (**a**) LSV curves recorded at a scan rate of 1.0 mV s−1 and (**b**) Tafel plots of the CO-150, CO-300, and CO-450 electrode films measured in an alkaline KOH condition.

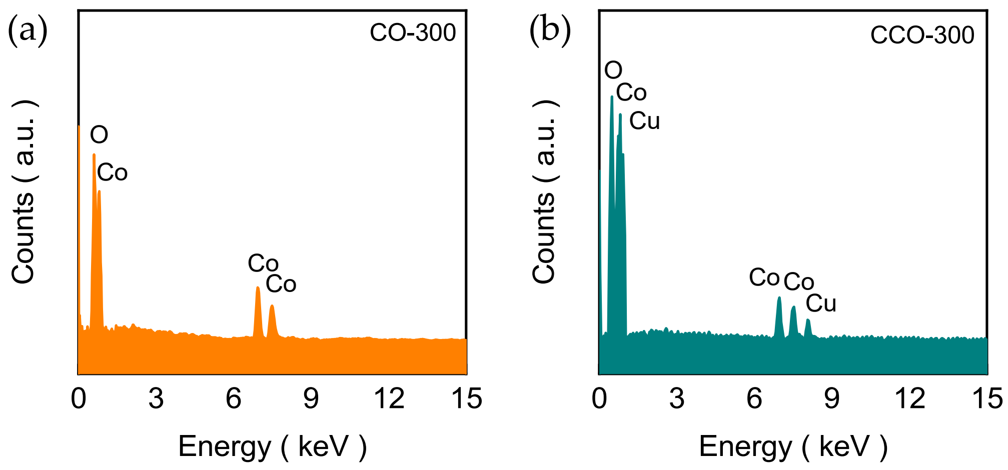

Figure S4. EDS spectra of the (**a**) CO-300 and (**b**) CCO-300 electrode films.

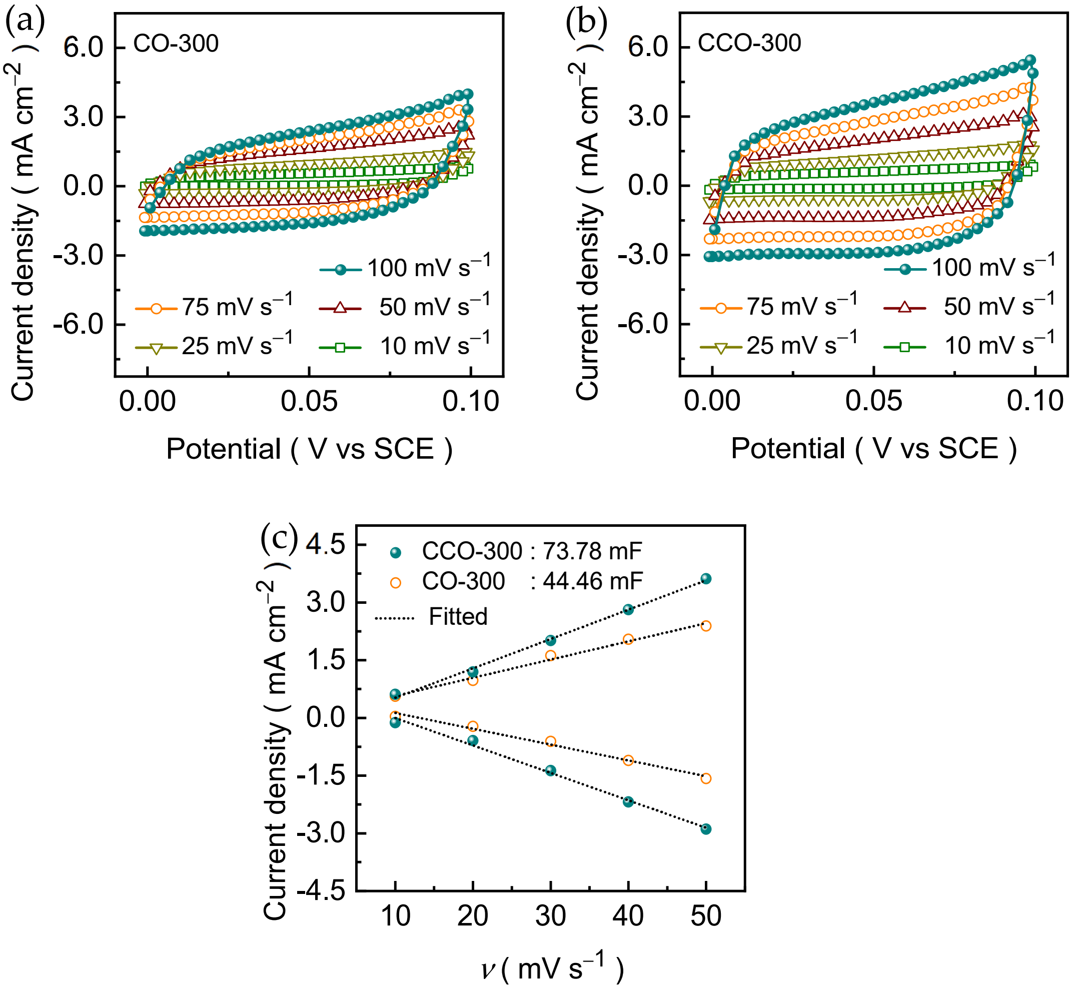

Figure S5. Non-Faradaic CV curves of the (**a**) CO-300 and (**b**) CCO-300 electrode films measured at different scan rates. (**c**) Non-Faradaic current densities as a function of scan rate obtained at 0.05 V (*vs*. SCE) to estimates the C_{DL} and *ECSA*.

The electrochemically active surface area (ECSA) of CO-300 and CCO-300 electrodes was evaluated by estimating the double-layer capacitance (C_{DL}) from non-faradaic CV measurements (Figure S5a,b). The CV curves were recorded within a potential window range free of Faradaic processes at varying scan rates, where the current response is dominated by double-layer charging. The capacitive current density (J_a and J_c), extracted from the anodic and cathodic currents at a fixed potential, shows a linear dependence on scan rate, and the average slope of this linear fit yields C_{DL}. The obtained C_{DL} values are 44.46 mF cm^{-2} for CO-300 and 73.78 mF cm^{-2} for CCO-300. Assuming a typical specific capacitance of 0.04 mF cm^{-2} for a smooth electrode surface in alkaline KOH medium, the corresponding *ECSA* values are estimated to be ~ 1111 cm^2 for CO-300 electrode and ~ 1845 cm^2 for CCO-300 electrode. The substantially higher *ECSA* of CCO-300 electrode confirms the increased density of electrochemically accessible active sites, consistent with its more open nanosheet architecture, and provides a clear structural basis for its enhanced OER activity and accelerated charge-transfer kinetics.

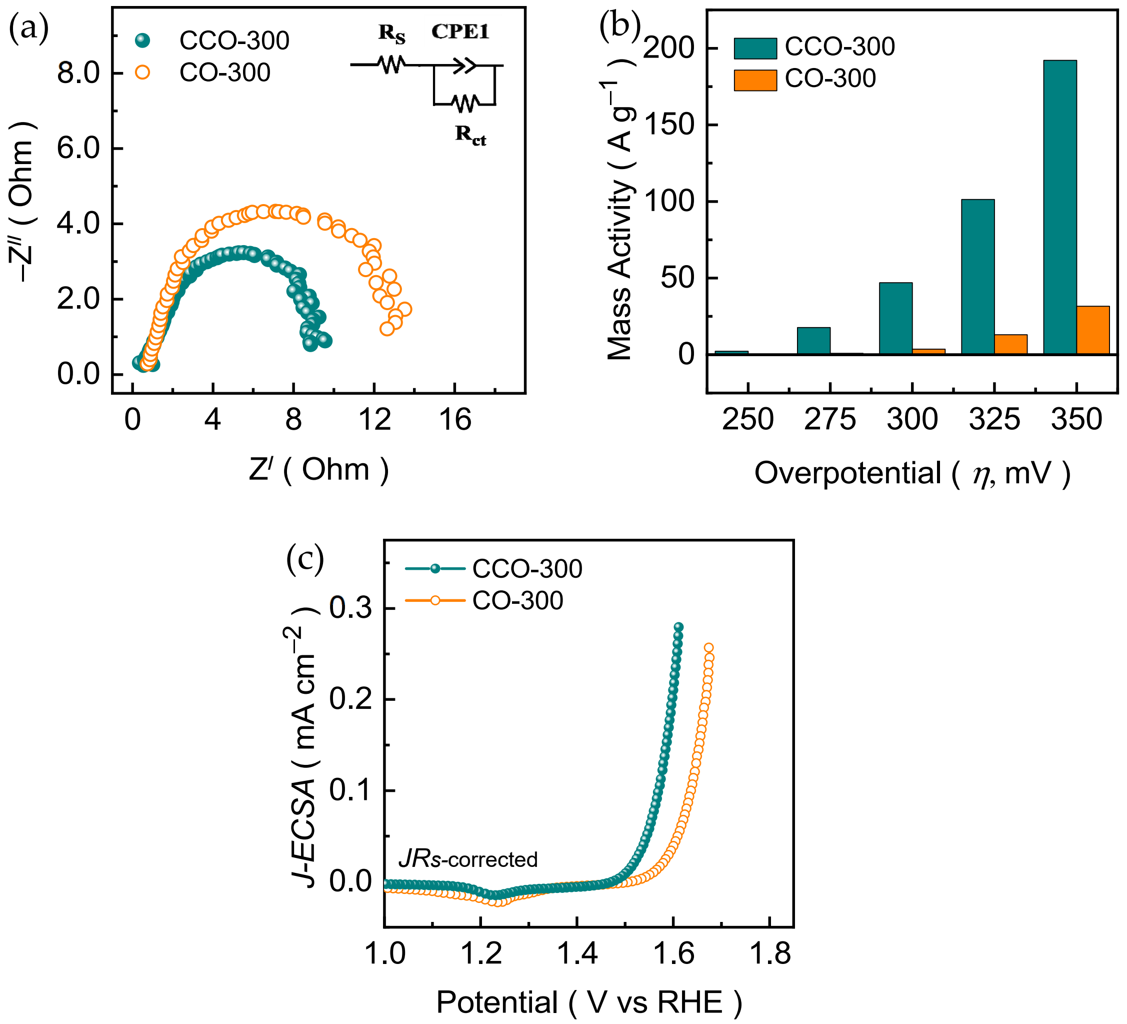

Figure S6. (**a**) Nyquist impedance curves along with the tank circuit, (**b**) Mass activity plots, and (**c**) *ECSA*-normalized LSV curves for the CO-300 and CCO-300 electrode films.

Table S2. Fitted electrochemical EIS parameters for the CO-300 and CCO-300 electrodes obtained from Nyquist plots using the equivalent circuit model shown in Figure S6a. The table summarizes the solution resistance (*R*s), charge-transfer resistance (*R*ct), and constant phase element (CPE) parameters extracted from nonlinear least-squares fitting. All EIS measurements were performed in 1.0 M KOH under identical conditions.

No.	Catalyst film	*R*s (Ω)	*R*ct (Ω)	*CPE* ($\Omega^{-1} \cdot s^n$)
1	CO-300	0.631	12.513	0.0037
2	CCO-300	0.597	8.504	0.0035
2	CCO-300 after stability	0.642	8.717	0.0034

Electrochemical impedance spectroscopy (EIS) was conducted to evaluate the interfacial charge-transfer behavior of the CO-300 and CCO-300 catalyst films. The Nyquist plots display a single, quasi-semicircular feature in the high frequency region, characteristic of a charge-transfer-controlled process at the electrode-electrolyte interface. The solution resistance *Rs* values are almost comparable for CO-300 (0.631 Ω) and CCO-300 (0.597 Ω) electrodes, indicating similar electrolyte resistance and electrical contact with the Ni foam substrate. In contrast, CCO-300 electrode exhibits a substantially lower charge-transfer resistance (8.504 Ω) than CO-300 (12.513 Ω), reflecting the enhanced interfacial electron-transfer kinetics induced by Cu incorporation. After stability testing, CCO-300 electrode exhibits insignificant change in the charge-transfer resistance value, confirming the structural robustness and sustained charge-transport efficiency during prolonged electrolysis.

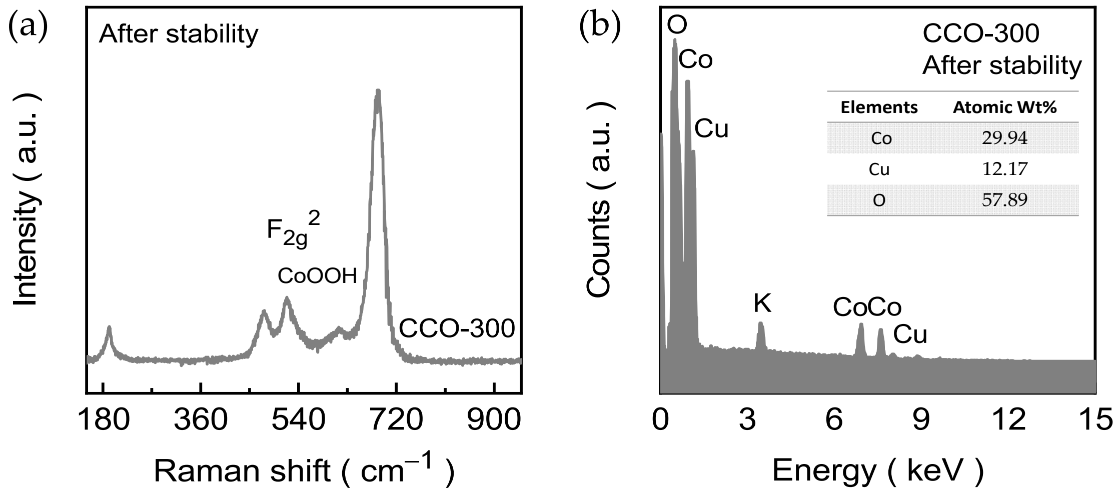

Figure S7. Post-stability measured (**a**) Raman and (**b**) EDS spectrum for the CCO-300 electrode film.

Supporting References

1. Zou, X.; Liu, Y.; Li, G.-D.; Wu, Y.; Liu, D.-P.; Li, W.; Li, H.-W.; Wang, D.; Zhang, Y.; Zou, X., Ultrafast Formation of Amorphous Bimetallic Hydroxide Films on 3D Conductive Sulfide Nanoarrays for Large-Current-Density Oxygen Evolution Electrocatalysis. *Adv. Mater.* **2017,** *29* (22), 1700404.
2. Dong, Q.; Wen, B.; Zhao, X.; Wang, P.; Lyu, X., Electronic structure tailoring of $CuCo_2O_4$ for boosting oxygen evolution reaction. *Separation and Purification Technology* **2025,** *353*, 128552.
3. Hoang, T. T. H.; Gewirth, A. A., High Activity Oxygen Evolution Reaction Catalysts from Additive-Controlled Electrodeposited Ni and NiFe Films. *ACS Catal.* **2016,** *6* (2), 1159-1164.
4. Dong, Q.; Zang, L.; Sun, D.; Xu, Y.; Zhao, D.; Lyu, X., Sulfur-doping assisted defective $CuCo_2O_4$ as a bifunctional electrocatalyst for efficient water splitting. *J. Colloid Interface Sci.* **2025,** *698*, 138053.
5. Peugeot, A.; Creissen, C. E.; Karapinar, D.; Tran, H. N.; Schreiber, M.; Fontecave, M., Benchmarking of oxygen evolution catalysts on porous nickel supports. *Joule* **2021,** *5* (5), 1281-1300.
6. Cheng, X.; Lei, C.; Yang, J.; Yang, B.; Li, Z.; Lu, J.; Zhang, X.; Lei, L.; Hou, Y.; Ostrikov, K., Efficient Electrocatalytic Oxygen Evolution at Extremely High Current Density over 3D Ultrasmall Zero-Valent Iron-Coupled Nickel Sulfide Nanosheets. *ChemElectroChem* **2018,** *5* (24), 3866-3872.
7. Shi, W.; Zhang, Y.; Bo, L.; Guan, X.; Wang, Y.; Tong, J., Ce-Substituted Spinel $CuCo_2O_4$ Quantum Dots with High Oxygen Vacancies and Greatly Improved Electrocatalytic Activity for Oxygen Evolution Reaction. *Inorg. Chem.* **2021,** *60* (24), 19136-19144.
8. Meena, A.; Abu Talha Aqueel, A.; Aditya Narayan, S.; Vijaya Gopalan, S.; Im, H.; Cho, S., Highly Efficient CoFeP Nanoparticle Catalysts for Superior Oxygen Evolution Reaction Performance. *Nanomaterials* **2024,** *14* (17), 1384.
9. Lu, X.; Zhao, C., Electrodeposition of hierarchically structured three-dimensional nickel–iron electrodes for efficient oxygen evolution at high current densities. *Nat. Commun.* **2015,** *6* (1), 6616.
10. Ding, X.; Liu, J.; Cang, R.; Chang, X.; Zhang, M., Electrospun Hollow Carbon Nanofibers Decorated with $CuCo_2O_4$ Nanowires for Oxygen Evolution Reaction. *Catalysts* **2022,** *12* (8), 851.
11. Talha Aqueel Ahmed, A.; Ho Lee, C.; Saad Ansari, A.; Pawar, S. M.; Han, J.; Park, S.; Shin, G.; Yeon, S.; Cho, S.; Seol, J.; Uck Lee, S.; Kim, H.; Im, H., Hybridized heterostructure of CoS and MoS_2 nanoparticles for highly-efficient and robust bifunctional water electrolysis. *Appl. Surf. Sci.* **2022,** *592*, 153196.
12. Guo, D.; Qi, J.; Zhang, W.; Cao, R., Surface Electrochemical Modification of a Nickel Substrate to Prepare a NiFe-based Electrode for Water Oxidation. *ChemSusChem* **2017,** *10* (2), 394-400.

13. Tian, J.; Wang, Z.; Wang, Y.; Yuan, D.; Tian, F.; Zhang, L., Nitrogen-doped Binary Spinel $CuCo_2O_4$/C Nanocomposite: An Efficient Electrocatalyst for Oxygen Evolution Reaction. *ChemNanoMat* **2020,** *6* (11), 1652-1657.
